# Public-private delivery of insecticide-treated nets: a voucher scheme in Volta Region, Ghana

**DOI:** 10.1186/1475-2875-6-14

**Published:** 2007-02-02

**Authors:** Margaret Kweku, Jayne Webster, Ian Taylor, Susan Burns, McDamien Dedzo

**Affiliations:** 1 Ghana Health Service, Volta Regional Health Directorate, P.O. Box HP 72, Ho. Volta Region, Ghana; 2 Gates Malaria Programme, London School of Hygiene and Tropical Medicine, Keppel Street, London WC1E 7HT, UK; 3 TARGETS Consortium, London School of Hygiene and Tropical Medicine, Keppel Street, London WC1E 7HT, UK; 4 Exp Momentum, Box AT65, Achimota, Accra, Ghana

## Abstract

**Background:**

Coverage of vulnerable groups with insecticide-treated nets (ITNs) in Ghana, as in the majority of countries of sub-Saharan Africa is currently low. A voucher scheme was introduced in Volta Region as a possible sustainable delivery system for increasing this coverage through scale-up to other regions. Successful scale-up of public health interventions depends upon optimal delivery processes but operational research for delivery processes in large-scale implementation has been inadequate.

**Methods:**

A simple tool was developed to monitor numbers of vouchers given to each health facility, numbers issued to pregnant women by the health staff, and numbers redeemed by the distributors back to the management agent. Three rounds of interviews were undertaken with health facility staff, retailers and pregnant women who had attended antenatal clinic (ANC).

**Results:**

During the one year pilot 25,926 vouchers were issued to eligible women from clinics, which equates to 50.7% of the 51,658 ANC registrants during this time period. Of the vouchers issued 66.7% were redeemed by distributors back to the management agent. Initially, non-issuing of vouchers to pregnant women was mainly due to eligibility criteria imposed by the midwives; later in the year it was due to decisions of the pregnant women, and supply constraints. These in turn were heavily influenced by factors external to the programme: current household ownership of nets, competing ITN delivery strategies, and competition for the limited number of ITNs available in the country from major urban areas of other regions.

**Conclusion:**

Both issuing and redemption of vouchers should be monitored as factors assumed to influence voucher redemption had an influence on issuing, and vice versa. More evidence is needed on how specific contextual factors influence the success of voucher schemes and other models of delivery of ITNs. Such an evidence base will facilitate optimal strategic decision making so that the delivery model with the best probability of success within a given context is implemented. Rigorous monitoring has an important role to play in the successful scaling-up of delivery of effective public health interventions.

## Background

Although insecticide-treated nets (ITNs) are a proven effective intervention for reducing child morbidity and mortality, coverage is still low across sub-Saharan Africa [1,2]. There is a recognized need to scale-up coverage of ITNs and other proven effective interventions in order to reach the Millennium Development Goal (MDG) of reducing child mortality by 50% by 2015 [3]. ITNs have also proven to have a beneficial impact on pregnancy outcome in malaria endemic regions of sub-Saharan Africa [4]. Targets of 60% of children under five years of age, and 60% of pregnant women sleeping under ITNs by 2005 were set at the Heads of States meeting in Abuja in 2000 [5]. Although the majority of countries have not yet reached these goals, more recent increased targets of 80% coverage of target groups have been suggested [6]. Countries are using a variety of strategies in their quest to reach these goals [1] but evidence-based support for strategic decision making on which system to implement is lacking.

Sustained universal provision of ITNs to pregnant women at risk of malaria, through antenatal clinics (ANC) should be a priority [7] as this will allow protection of the mother, the unborn child and the infant as mothers and their newborn children share sleeping places. Delivery of ITNs to pregnant women through ANC may involve direct provision of the ITN [8], either with purely public sector input [9], or with assistance from the private sector in the form of NGOs [10,11]. Alternatively, subsidies may be delivered at ANC and the product collected from the retail sector, via voucher schemes [12-14].

The National Malaria Control Programme in Ghana has a history of innovation in the delivery of ITNs, often leading the way with new delivery systems [15-18]. These have however, been on a relatively small scale with limited funding and, therefore, have not had a significant impact on coverage at the national level. The latest nationally representative estimates of use of ITNs amongst target groups are 2.7% for pregnant women and 3.5% for children under 5 years of age [19]. An ITN voucher scheme was seen as a promising way forward in sustainable scale-up of ITNs in Ghana, with a pilot scheme in two regions of the country and plans to scale-up to other regions if successful. Voucher schemes use a mixed public-private sector approach, with subsidies delivered through the public sector and the ITNs through the private commercial sector.

The inadequate focus on operational research for large-scale implementation has recently been outlined [20] and a number of possible reasons for this suggested. Operational research and programme monitoring and evaluation are closely linked. For many programme implementers, evaluation through household surveys is prohibitively expensive and technically complex, and therefore outcome level evaluation of the programme is beyond capacity. Programme monitoring is generally used to provide feedback on planned programme activities and to modify any actions that are proving ineffective and inefficient during implementation. Monitoring methods tend to be less expensive and technically complex than those of evaluations and are therefore usually within the financial and technical capacity of most programme implementation teams. The example of the pilot ITN voucher scheme in Volta Region Ghana is used to highlight the importance of monitoring data not only for adjusting implementation but also for providing evidence on the barriers to scaling-up of ITNs.

## Methods

### Study setting

In an attempt to scale-up coverage with ITNs in Ghana support was given to the design, implementation, and monitoring and evaluation of a voucher scheme in two pilot regions: Volta Region and Eastern Region. The Volta Region pilot began eight months ahead of that of the Eastern Region, and is the focus of this paper. Volta Region lies to the extreme east of the country and shares a border with Togo. Like Ghana as a whole, Volta Region has three ecological zones with grassland along the southern coast, semi-deciduous forest in the central zone and semi-Savannah in the North. The central and southern zones have two wet seasons, the major one from May to July and the minor one from September to November. The north of the region has one wet season from May to August. The population of Volta Region is 1.6 million according to the 2000 population census. Of the ten regions in Ghana, Volta Region has the highest household ownership of mosquito nets at 46.1%, however ownership of ITNs is much lower at 2.5% [19].

Volta Region has a total of 285 health facilities, 203 of which are administered by the Ghana Health Service (GHS). Amongst these GHS facilities there are 1 regional and 9 district hospitals, 1 polyclinic, 143 health centres and 49 clinics. The voucher scheme was implemented in all GHS and Mission health facilities within the region which had a midwife and/or antenatal clinic (ANC).

### The structure of the voucher scheme

The scheme was designed such that discount vouchers are given to pregnant women during their first presentation at antenatal clinic. The voucher entitles the recipient to ¢40,000 (approximately US$4.20) discount on an ITN available through retail outlets. The recipient or their representative takes the voucher to a retail outlet stocking approved ITNs and provides the top-up cash required, together with the voucher, for an ITN. The retailer removes a 'proof-of-purchase' sticker from the ITN packaging as it is sold and attaches the sticker to the voucher. The retailer is then able to exchange the voucher for more stock from his distributor, and keeps the top-up value of cash from the client. The distributor then exchanges the voucher with its proof-of-purchase sticker attached for cash from a management agent. Vouchers presented to the management agent without a proof-of-purchase sticker are rejected.

The management agent is responsible for redeeming vouchers, for supplying vouchers to the health facilities, and for monitoring of voucher supplies and redemptions. During the one year pilot in Volta Region there were three approved ITNs which were PermaNet, Dawanet and K-O Net. PermaNet is a Long Lasting Insecticidal Net (LLIN), Dawanet is mosquito net that is factory-treated with insecticide, and K-O Net is an untreated net packaged (bundled) with an insecticide treatment kit. These were delivered to retail outlets in the region initially by two distributors, Transcol Ltd and AgriMat. During the last three months of the pilot they were joined by a third distributor NetCo Rockville.

Two distinct monitoring activities were undertaken during the one year pilot voucher scheme which were 1) monitoring of the voucher issuing and redemption and 2) stakeholder interviews with health facility staff, retailers involved in the voucher scheme, and pregnant women exiting health facilities.

### Monitoring of voucher issue and redemption

The programmatic objective of voucher issue and redemption monitoring was to ensure adequate supply of vouchers to clinics and to quantify outputs of the pilot project. Vouchers were distributed to all health facilities with antenatal clinics in the region by the management agent (Exp Momentum). A simple tool was developed to monitor numbers of vouchers given to each health facility, numbers issued by the facility, and numbers redeemed by distributors. Monitoring of distribution and issuing of vouchers was conducted at the health facility and redemptions at the central level. In order to aid in monitoring, the vouchers have a serial number by which they can be matched to the issuing health facility. The health facilities were visited monthly by the management agent to replenish vouchers and conduct monitoring activities. ANC attendance data by district was gathered from quarterly reports submitted to regional level.

Assuming that all vouchers issued from ANC were given to pregnant women, the proportion of registrants issued with a voucher was calculated as the number of vouchers issued, divided by the number of ANC registrants. The proportion of registrants issued with a voucher was calculated in this way at the regional, zonal and district levels. The proportion of vouchers redeemed was calculated as the number of vouchers redeemed for cash by the management agent divided by the number of vouchers issued at the regional, zonal and district levels. Linear regression was used to compare the proportion of vouchers redeemed that were issued from urban and rural clinics. Lack of individual health facility attendance data meant that it was not possible to compare the proportion of vouchers issued from urban and rural clinics.

### Stakeholder interviews

Interviews were undertaken to identify any problems occurring during the pilot of the voucher scheme at the ANC and retail levels as perceived by ANC staff, retailers and pregnant women; and to document findings and recommend adjustments to the pilot project. Semi-structured questionnaires were developed in order to address questions at the a) health facility, b) retail and c) household levels.

Interviews were conducted by the Volta Regional Health Directorate Research Team (VRHDRT), district disease control officers, and some National Service Personnel. Three rounds of interviews were undertaken in all eleven districts, three months, six months and twelve months post implementation of the scheme. All district hospitals, both government and mission, were included in the sample, together with one health centre with ANC from each sub-district. In hospitals, two ANC staff involved in voucher distribution was interviewed. At the sub-district level, in each health facility, all midwives involved in the provision of ANC services were interviewed (most health centres have only one midwife – with a maximum of two). Retailers within the catchment area of the ANC clinics were interviewed. Advice on where to find the nearest retail outlet was sought from the clinic staff; this was not therefore a census of retail outlets, but was aimed specifically at outlets known to be involved in the voucher scheme. If there were traders selling nets outside the clinic – the retail outlet from which they came was selected. Data collectors started with retail outlets close to the district hospital plus retail outlets close to 1 health centre per sub-district. At the hospital level three first time attendees (registrants) and three multiple visit attendees were sought for interview. Numbers of interviews conducted with ANC staff, retailers, ANC registrants and multiple attendees are presented in Table 1. Data from interviews with health facility staff, retailers and pregnant women was collated and analysed by themes based around 1) uptake of vouchers from health facilities and 2) exchange of vouchers for ITNs in retail outlets.

**Table 1:** Number of respondents during each round of interviews

Number of months post implementation	Number of respondents			
	
	Health facility staff	Retailers	ANC registrants	ANC multiple attendees
3 months	92	58	89	107
6 months	69	58	89	107
12 months	67	58	80	83

The monitoring activities were approved by the Ghana Health Service Ethical Review Committee, and the ethical committee of the London School of Hygiene and Tropical Medicine.

## Results

### Voucher issuing and redemptions

Vouchers were issued to 133 health facilities across the region (Table 2), of these 40 were in urban areas and 93 rural (urban clinics were defined as those with a catchment population ≥ 5,000, and rural with < 5,000). During the one year pilot 43,050 vouchers were provided to the clinics across the region, 60% of these (25,926) were issued to eligible women from the clinics. This equates to 50.7% of the 51,658 registrants being provided with a subsidy towards an ITN (Table 3). Of the vouchers issued from the clinics 66.9% (17,888) were redeemed by distributors back to the Management Agent.

**Table 2:** Health facilities involved in the voucher scheme by district

Zone	District	**Population**	No facilities		
			
			Total	Urban	Rural
**Northern**	Krachi	172,430	11	2	9
	Kadjebi	56,064	7	3	4
	Jasikan	119,987	8	3	5
	**Zonal total**		**26**	**8**	**18**
**Central**	Hohoe	165,014	24	3	21
	Kpando	121,794	18	4	14
	Ho	253,732	31	8	23
	North Tongu	140,584	9	3	6
	**Zonal total**		**82**	**18**	**64**
**Southern**	Akatsi	100,786	5	2	3
	South Tongu	69,879	3	3	0
	Ketu	255,813	10	3	7
	Keta	144,112	7	6	1
	**Zonal total**		**25**	**14**	**11**

Total			**133**	**40**	**93**

**Table 3:** Vouchers issued and redeemed by zone and district during the 12 month pilot

	**District**	**Expected no. pregnancies (4% total population^a^)**	**No. registrants April '04 – March '05**	**No. vouchers issued**	**Proportion registrants issued with voucher^b^(%)**	**No. vouchers redeemed**	**Proportion vouchers redeemed^c^(%)**
**Northern**	Krachi	6,897	5,376	665	12.4	393	59.1
	Kadjebi	2,243	2,861	1,728	60.4	976	56.5
	Jasikan	4,799	4,898	2,618	53.5	1,478	56.5
	**Total**	**13,939**	**13,135**	**5,011**	**38.1**	**2,847**	**56.8**

**Central**	Hohoe	6,601	4,064	3,973	97.8	2,250	56.6
	Kpando	4,872	5,105	3,327	65.2	2,717	81.7
	Ho	10,149	6,077	4,850	79.8	3,196	65.9
	North Tongu	5,623	5,298	2,349	44.3	1,777	75.6
	**Total**	**27,254**	**20,544**	**14,499**	**70.6**	**9,940**	**68.6**

**Southern**	Akatsi	4,031	2,100	1,170	55.7	655	56.0
	South Tongu	2,795	2,257	1,069	47.4	801	74.9
	Ketu	10,233	8,019	2,659	33.2	2,035	76.5
	Keta	5,764	5,603	1,788	31.9	1,260	70.5
	**Total**	**22,823**	**17,979**	**6,686**	**37.2**	**4,751**	**71.1**

Total		**64,0168**	**51,658**	**26,196**	**50.7**	**17,538**	**66.9**

^a ^Source 2000 census

^b ^No. of vouchers issued divided by no. of registrants

^c ^No. of vouchers redeemed divided by no. of vouchers issued (Redemption Rate)

Using routine health facility data the issuing of 14,499 vouchers from the central zone of the region suggests that 70.6% of registrants were issued with a voucher, compared with 38.1% and 37.2% in the northern and southern zones, respectively. Variation in proportion of vouchers redeemed across the zones was less pronounced at 56.8%, 68.6%, and 71.1%, in northern, central and southern zones respectively. There was wide disparity in the proportion of registrants issued with vouchers across districts of the region varying from 12.4% in Krachi to 97.8% in Hohoe. The proportion of vouchers issued from clinics that were redeemed by distributors back to the Management Agent, varied from 56.0% in Akatsi to 77.5% in Kpando.

Of the 133 health facilities provided with vouchers, 11 did not issue any to registrants, this included 3/31 facilities from Ho, 3/24 facilities from Hohoe and 5/11 facilities in Krachi. Ten of these non-issuing facilities were rural, the one urban facility being in Ho. Nearly two thirds (63.3%) of vouchers issued from urban health facilities were redeemed compared with 47% of those issued from rural clinics (p = 0.009). With the exception of South Tongu, variation in the proportion of vouchers redeemed between health facilities was greater than variation between districts that is, intra-district variation was greater than inter-district variation. In Ho district, a mean of 65.9% of vouchers issued from health facilities were redeemed across the district, however, the median proportion redeemed by facilities was 49.7% ranging from 7.1% to 100%.

### Process issues influencing uptake of vouchers from ANC

Process issues identified through interviews were divided into those relating to voucher uptake from ANC and exchange of vouchers for ITNs in retail outlets. Issues affecting uptake of vouchers from health facilities were categorised as those relating to health facility staff and those relating to pregnant women. The major health facility staff issues influencing uptake of the vouchers were eligibility criteria, direct selling of ITNs from health facilities, non-availability of ITNs and voucher stock-outs.

During each of the three rounds of interviews a majority of health staff said that they had experience of eligible women leaving ANC without a voucher. At three months post implementation this was the response of 75% (69/92) of health staff, at six months 61% (42/69) and at 12 months 67% (45/67). The reasons for this and their relative importance changed through the one year period. At three months post implementation the reason why most women left without a voucher was due to a decision of the health staff. Fifty percent of all staff interviewed had decided not to give a woman a voucher because she was '*not ready with money*', that is, she was not ready to pay the top-up fee. After six months of implementing the voucher scheme the reasons for non-issuing were diverse, but the main reasons were identified as the woman already having a net 24% (10/42), and that there were no vouchers available 24% (10/42). After one year of implementation the major reasons stated for women leaving ANC without vouchers was that the '*women have a net already*' 36% (16/45), '*women cannot afford the top-up*' 20% (9/45), and '*there were no vouchers*' 13% (6/45). At both the six month and 12 month stages of implementation the main reasons for non-issuing of vouchers were no longer due to decisions of the midwife, but were either decisions of the pregnant woman or lack of supplies.

The numbers of women who said that they were offered and accepted a voucher over the three rounds of survey fluctuated: 43% (33/89) of registrants and 53% of multiple attendees (57/107) at 3 months post implementation; 79% (55/70) registrants and 83% (63/76) multiple attendees at six months post implementation; and 67% (54/80) registrants and 41% (34/83) multiple attendees at 12 months post implementation. The peak in issuing of vouchers was at six months post implementation.

### Voucher exchange

At the 12 month post implementation point 75% (53/71) of the retailers interviewed had less than 12 months experience in selling nets (treated or untreated). The main problem faced by retailers with respect to the voucher scheme was inadequate stock of ITNs. At the three month post implementation survey the maximum stock of 53% (31/58) of retailers was between five to 20 ITNs, 28% (16/58) had between 21 to 40 and 17% (10/58) had 41 to 60. At six months post implementation stock levels were very similar to three month levels, with only 4 retailers reporting stocks ever increasing beyond 60 ITNs. By the one year post-implementation survey, although stocking levels had increased with 20% (12/58) of retailers now reporting that their stock levels had ever reached above 60 ITNs, 80% (46/58) reported that they had stock-outs of ITNs since joining the voucher scheme.

Retailers were asked about the influence of the voucher scheme on their business. Three months post-implementation, 33% (19/58) of retailers interviewed said that their business had changed due to the voucher scheme, at six months post-implementation this was the response of 47% (27/58) of respondents and at 12 months 59% (34/58). When questioned further on the nature of their ITN business 34% (20/58) of respondents in the first survey said that they only sold ITNs to voucher customers, this decreased to 25% (15/58) in the second survey and 17% (10/58) after 12 months of implementation.

Seventy percent of women who had been given a voucher at ANC had already bought the ITN three months post implementation of the scheme, six months post-implementation 51% of those given vouchers had bought, and 12 months post-implementation 63% had bought. Reasons for not having bought concerned non-availability of ITNs or a particular type of ITN, non-availability of money, already owning a net and perceptions that there was no current need of a net.

The majority of women (88%, 97% and 94% at three month, six month and 12 month post-implementation surveys, respectively) exchanged their voucher for an ITN in the place to which they were directed by the midwife who issued the voucher. Over the 12 months of the pilot the proportion of those who had bought ITNs with a voucher who reported that they had some choice in the size, shape or colour increased from 58% (23/40) at three months post implementation to 74% (23/31) at 12 months. Relatively few pregnant women reported that they had a choice of brands the greatest proportion being 19% (6/31) at 12 months post implementation.

## Discussion

**Figure 1:**
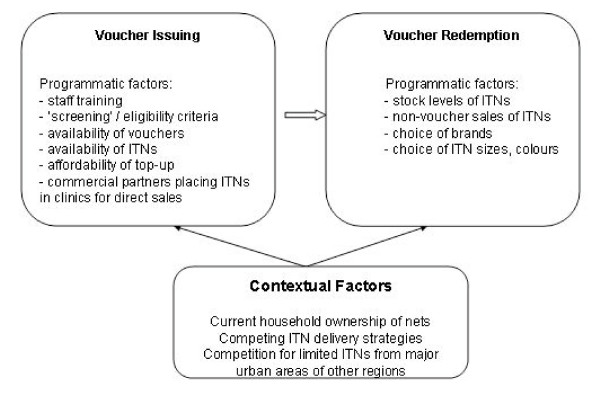
Factors affecting voucher issuing and redemption in the 12-month pilot ITN voucher scheme.

Delivery of ITNs through voucher schemes involves five key transactions [8]. In Volta Region, monitoring activities focussed on two points amongst these five transactions, thereby dividing them into two groups of activities. The first group, voucher uptake or issuing, included distribution of vouchers to health facilities and their delivery by midwives in ANC to pregnant women; the second group of activities, voucher redemption, included exchange of vouchers for ITNs in retail outlets by pregnant women, retailer redemption of the voucher for more stock from the distributor, and distributor redemption of the voucher for cash from the management agent. Within the mixed public-private sector delivery of ITNs through voucher schemes voucher uptake is predominantly the responsibility of the public sector and voucher redemption, of the private sector. The major factors affecting voucher issue and redemption in Volta Region are summarised in Figure 1. The findings emphasize the links between the two sectors with availability of ITNs in the private sector influencing public sector delivery of the subsidy, and actions and choices made by health staff influencing the growth of the private sector.

Voucher issuing records together with routine attendance data suggest that over the one year period, approximately half of all eligible women attending ANC were issued with a voucher. Voucher uptake varied geographically by zone, district and health facility and interviews suggest that reasons for this variable uptake changed over the one year period. Nearly twice as many pregnant women attending ANC in the central zone of the region took a voucher compared to those in the northern and southern zones. Findings from a household survey in Malawi show a similar pattern of district level variation at the outcome level with use of ITNs by pregnant women varying from 3.5% to 69.6% across districts [21]. The major delivery system for ITNs in Malawi is the public-private delivery of direct product through ANC with the support of Population Services International (PSI). In Volta Region, nearly all of those attending ANC were issued with a voucher in Hohoe district, contrasting with only 12% of those from Krachi district. Krachi district is particularly hard to access; it is separated from other districts in the region by the Volta Lake. Access within the district is also difficult with many areas requiring a boat.

Voucher redemption was high. Two thirds of the vouchers issued had been redeemed back to the management agent. As with voucher issuing, redemption varied between zones, districts and facilities, but the variation was less pronounced. However, a significantly greater proportion of vouchers issued from urban health facilities were redeemed compared to those issued from rural health facilities. The high voucher redemption rates over the one year period compared to the voucher issuing rates suggest that most of the decision making on whether women were going to use a voucher to buy an ITN was at the voucher issuing stage. Those who were not likely to buy (or whom the midwife felt was not likely to buy), did not take (or were not offered) a voucher. The majority of those who took a voucher intended to use it to buy an ITN.

With the monitoring methods used it is likely that we have overestimated the voucher issuing rate and underestimated the voucher redemption rate to retailers of ITNs. The assumption that all vouchers were issued to eligible women at ANC may be optimistic as this assumes perfect targeting of the subsidy. Evidence to date from other ITN voucher schemes [22,23], and from direct delivery of ITNs to pregnant women through ANC [9] suggests that loss may be high within the public sector. It is likely that the proportion of women with vouchers, who used them to purchase an ITN, as calculated, is an underestimate because a number of these exchanged vouchers would still be with the retailers or the distributors, and not have completed the process of return to the management agent.

Interviews with health facility staff and with pregnant women suggest that the major reasons for women not taking a voucher varied over the one year period. Initially, midwives did not offer a voucher to all pregnant women, unless they could show that they were able to pay the top-up amount required to buy the ITN. This 'screening' or imposition of eligibility criteria has been noted in other voucher schemes in Senegal [14] and in Zambia [24]. Knowledge of issues encountered in previous voucher schemes meant that non-imposition of eligibility criteria was stressed during the training programme in Volta Region. However, problems in attendance at training sessions reduced the effectiveness of the messages. Dissemination of monitoring findings four months into the scheme prompted further training and strengthened supervision, resulting in a marked decrease in the imposition of eligibility criteria as assessed during monitoring at 6 and 12 months into the scheme.

Four districts of the region (Nkwanta, Hohoe, North Tongu and Keta) had previously been supplied with ITNs from the National Malaria Control Programme (NMCP) or District Health Management Teams to be sold for ¢20,000 to pregnant women and to children under five years of age from health facilities. With the advent of the pilot voucher scheme a policy decision was taken by the regional health directorate together with the NMCP that no more ITNs would be supplied to health facilities for sale due to the risk that this scheme would directly compete with the voucher scheme. Interviews with health staff highlighted two issues: firstly several health facilities were still selling ITNs after the start of the voucher scheme from distributions of ITNs that they had received previously. Secondly, commercial partners had approached midwives to sell ITNs directly from health facilities rather than sending clients to retail outlets with their vouchers. Selling of commercial sector ITNs directly from health facilities meant that both the voucher and the ITN were delivered in health facilities to the exclusion of commercial retailers.

During the later half of the pilot the main reasons why women did not take or were not offered a voucher involved lack of ITNs, lack of vouchers, and already owning a net. The first two of these are supply issues, but may also involve decisions by the midwife; the third is a direct decision by the pregnant woman.

Almost half (46.1%) [19] of all households in Volta Region own a mosquito net, consequently almost half of all women offered a voucher will already have a net in their household. Very few of the nets already in households in Volta Region are treated; untreated nets are approximately 50% as protective as treated nets [25-28]. Buying a new ITN would, therefore, provide extra protection to the pregnant woman. It is unclear whether the main barrier to buying a new ITN is the cost of the top-up to the voucher subsidy, or preferences for different kinds of nets. However, recent experiences from Niger suggest that people who own nets may not be willing to replace these with ITNs even when they are given free of charge. The linked polio and ITN campaign in Niger distributed ITNs to 90% of families with a child under five years of age [29] within the context of an existing 62% household ownership of nets [30]. Use of the ITNs by children under 5 years of age, one month post campaign was only 15.4% [31]. This may be contrasted with an ITN voucher scheme in Tanzania where 94% of the ITNs bought with vouchers were used by the target group [22]. Non-uptake of vouchers by those pregnant women who do not intend to use an ITN avoids wastage of the value of the subsidy. Escalated information and education is needed to convince those without nets on the importance of sleeping under an ITN during their pregnancy. For those women who already have a net (particularly where these nets are locally made and therefore different to ITNs) alternative strategies may be needed to ensure that they sleep under an ITN. In the context of high household ownership of nets, strategies are needed to compliment delivery of ITNs through ANC (whether direct, via voucher, free or subsidised) to convert existing nets to ITNs. In areas of high coverage with nets, (re)treatment campaigns may be the most effective strategy for ensuring that the target group sleep under an ITN.

At the voucher issuing stage, 'lack of ITNs' as a reason for pregnant women not taking a voucher was mainly a decision of the midwife. Midwives were trained to direct pregnant women to outlets where they could exchange the voucher for an ITN. When the midwives knew, or perceived, that there were no ITNs available in the outlets within the vicinity of the health facility, they did not offer a voucher. Lack of ITNs was a constant problem in some geographic areas as the commercial sector was not well developed when the voucher scheme began. However, in other areas the commercial partners were able to increase the numbers of outlets supplying ITNs. The addition of a third distributor in the region whose business was established in response to the voucher scheme contributed to increased momentum from the commercial sector.

In the Volta Region pilot voucher scheme, the proportion of women who were issued vouchers that used them to buy a net was high. It is likely that this was influenced by the fact that most of those who did not intend to buy an ITN did not take a voucher. Whether those who bought an ITN use it cannot be assessed using programme monitoring but will be determined through a household survey.

Retailers' greatest concern about the voucher scheme as found during each round of interviews was low stock levels of ITNs. The retailers receive their ITNs from distributors, as mentioned above, the scheme started with just two distributors and increased to three. The financial capacity of each of these distributors was limited, as therefore was their capacity to allow long periods during which their capital was tied up. ITNs were given to retailers on credit and therefore the risks involved dictated initial small stock levels in the majority of outlets. Credit limits and penalties also limited the ability of distributors to reach 'hard-to-reach' areas where leaving large numbers of ITNs means tying up capital for long periods of time as sales are likely to be slow in such areas. Tying up of capital leaves the distributor liable to credit penalties. The alternative of visiting such areas regularly incurs prohibitive transport costs in terms of fuel and wear-and-tear of vehicles.

In the first few months of the voucher scheme some of the retail outlets, particularly those with low numbers of ITNs sold ITNs only to customers with vouchers. The voucher scheme aims to both provide a subsidy to the target group and to strengthen the retail sector with the aim of gradually reducing the need for the subsidy. Where ITNs are sold only through voucher sales the system is dependent upon donor money. Increasing non-voucher sales is one measure of the increasing strength of the private sector. Over the 12 month period an increasing number of retail outlets reported that they were selling to non-voucher customers.

Approximately seven months into the scheme, retail outlets in Volta Region suffered an acute shortage of ITNs. This was partly due to poor experience in forward planning by the distributors, and their limited financial capacity in terms of credit availability, but was significantly exacerbated by a limited duration voucher scheme project in the two largest urban areas of the country, Accra and Kumasi. The rapid sales available in these two urban areas were a much more attractive business proposition than the slower sales in the voucher pilot region, to the extent that the limited ITNs available in the country were diverted away from the Volta Region. At this point the management agent suspended supplies of vouchers to the clinics, and voucher scheme activities were severely impeded for a period of two months until supplies of ITNs were again introduced into retail outlets in the region.

This shortage of ITNs posed problems for the Volta Regional Health Directorate who in response to this shortage requested ITNs from the NMCP to fill this gap. ITNs were then supplied to all districts within the region to be sold to pregnant women from ANC.

Voucher schemes offer the potential for the private sector to take on the role of distribution and selling of ITNs thereby releasing public sector resources for other health interventions. Within this model the public sector direct their resources on delivery of the subsidy (voucher) to the target group together with advice and counselling on the use of ITNs. The findings from Volta Region highlight some of the ways in which defined processes may change during implementation due to decisions of the various actors. The impact of these delivery design changes needs further exploration in terms of coverage, equity of coverage and sustainability achieved.

Experiences during the one year pilot ITN voucher scheme in Volta Region outline the importance of monitoring of both the issuing and the redemption of vouchers. Factors that may be assumed to influence voucher redemption had an impact upon voucher issuing and vice versa. Coupling quantitative tracking of vouchers with qualitative interviews provided an insight into some of the reasons for initial low voucher issuing in some health facilities. Prompt dissemination of monitoring findings enabled programmatic adjustments to be undertaken speedily and effectively. Lessons were learnt which contributed to increased voucher issuing and redemptions in other regions as the programme scaled up. However in Volta Region, important factors affecting the success of the scheme were external to the programme and concerned the context within which the programme was working. More evidence is needed on how specific contextual factors influence the success of voucher schemes and other models of delivery of ITNs. Such an evidence base will facilitate optimal strategic decision making so that the delivery model with the best probability of success within a given context is implemented.

## Conflict of interests

Monitoring activities were funded by the Department for International Development, Ghana. Views presented in this paper do not necessarily represent the views of DFID. Exp Momentum is the management agent for the GFATM funded ITN voucher scheme in 3 further regions of Ghana.

## Authors' contributions

MK, JW and IT designed monitoring activities and analysed the data, all authors were involved in conceptualising the paper, JW wrote the first draft of the paper, MK, MD, SB and IT critically revised the manuscript
